# Recent Advancements in Imaging Techniques for Individual Extracellular Vesicles

**DOI:** 10.3390/molecules29245828

**Published:** 2024-12-10

**Authors:** Tatsuki Isogai, Koichiro M. Hirosawa, Kenichi G. N. Suzuki

**Affiliations:** 1 The United Graduate School of Agricultural Science, Gifu University, Gifu 501-1193, Japan; isogai.tatsuki.u2@s.gifu-u.ac.jp; 2 Institute for Glyco-Core Research (iGCORE), Gifu University, Gifu 501-1193, Japan; hirosawa.koichiro.g4@f.gifu-u.ac.jp; 3 Division of Advanced Bioimaging, National Cancer Center Research Institute (NCCRI), Tokyo 104-0045, Japan

**Keywords:** extracellular vesicles, single-particle tracking, total internal reflection fluorescence microscopy, dark-field imaging, super-resolution single-molecule localization microscopy

## Abstract

Extracellular vesicles (EVs), secreted from most cells, are small lipid membranes of vesicles of 30 to 1000 nm in diameter and contain nucleic acids, proteins, and intracellular organelles originating from donor cells. EVs play pivotal roles in intercellular communication, particularly in forming niches for cancer cell metastasis. However, EVs derived from donor cells exhibit significant heterogeneity, complicating the investigation of EV subtypes using ensemble averaging methods. In this context, we highlight recent studies that characterize individual EVs using advanced techniques, including single-fluorescent-particle tracking, single-metal-nanoparticle tracking, single-non-label-particle tracking, super-resolution microscopy, and atomic force microscopy. These techniques have facilitated high-throughput analyses of the properties of individual EV particles such as their sizes, compositions, and physical properties. Finally, we address the challenges that need to be resolved via single-particle (-molecule) imaging and super-resolution microscopy in future research.

## 1. Introduction

Cells recognize one another by exchanging substances, ultimately forming multicellular organisms. Extracellular vesicles (EVs) are small lipid membranes of vesicles ranging from approximately 30 to 1000 nm in diameter, secreted by nearly all cells of an organism. EVs were first identified in 1946 [1], with platelet-derived EVs being characterized in more detail in 1967 [2]. Initially referred to as “platelet dust”, they were considered to be a mechanism for expelling unwanted intracellular material. However, in 2007, it was discovered that EVs contain microRNAs (miRNAs) from secretory cells, which are internalized by recipient cells, regulating gene expression [3]. Since this discovery, EVs’ critical physiological functifons have been recognized. EVs carry nucleic acids, proteins, and even intracellular organelles such as mitochondria [4], which, once incorporated into recipient cells, alter their characteristics [5,6,7,8]. EVs have attracted significant attention as mediators of intercellular communication, and research in this field is rapidly advancing, covering varied disciplines. EVs are categorized into small EVs (sEVs) of 30~200 nm in diameter and microvesicles (MVs) of 100–1000 nm. sEVs are derived from multivesicular bodies (MVBs) and plasma membranes (PMs), while MVs are from PMs. EVs are typically isolated from cell culture medium using ultracentrifugation [9,10], size exclusion column [11], or an affinity-based method [12]. sEVs carry a variety of cargoes such as DNA, mRNA, miRNA, soluble proteins, membrane proteins, and lipids for cell–cell communications. For example, sEVs derived from cancer cells induce cell morphogenesis for angiogenesis [13], and sEVs derived from immune cells can activate the innate immune system [14]. On the other hand, since MVs are large (~1000 nm), MVs encapsulate large cellular organelles. For example, mesenchymal stem cells can transfer mitochondria to macrophages by secretion in MVs [15]. In addition, MVs containing mitochondria derived from monocytic cells are pro-inflammatory and induce type I interferons and tumor necrosis factor signaling in endothelial cells after uptake [16]. EVs have numerous potential applications [17], such as drug delivery [18], disease biomarkers [19], tissue regeneration [20], immune modulation [21], and stem cell therapy [22]. It has previously been reported that sEVs secreted by cancer cells are selectively internalized by cells in distant organs, establishing a pre-metastatic niche that creates an environment conducive to cancer cell metastasis [23,24]. The proteomic analysis of EVs has revealed that integrins α6β4 and α6β1 in EVs derived from 4175-LuT human breast cancer cells are associated with lung metastasis, while integrin αvβ5 in EVs from BxPC3 human pancreatic adenocarcinoma cells has been linked to liver metastasis [25]. However, it has become evident that variations in EV purification methods can lead to substantial differences in EVs’ membrane constituents and endosomal molecules. For example, it has been demonstrated that the constituents of EVs purified via the ultracentrifugation (pellet-down method) of cell culture supernatants differ significantly from those obtained via affinity purification using antibodies against marker proteins such as CD63 [12,26]. sEVs isolated by the affinity method using the TIM-4 protein that specifically binds to phosphatidylserine on the surface of EVs showed higher purity than those by ultracentrifugation [12]. Since the combination of ultracentrifugation, size exclusion chromatography, and affinity method is frequently employed, EV purification methods are so diverse that there are nearly as many approaches as there are publications discussing them. Given that purified EVs consist of a heterogeneous mixture of particles varying in size and originating from both the PM and intracellular multivesicular bodies (MVBs), the existence of distinct EV subtypes is plausible [27]. When analyzing EVs’ ensemble average, it is challenging to discern which characteristics belong to specific EV subtypes, thus hindering our understanding of their biological functions. Techniques such as Western blotting and flow cytometry with antibody beads capture only the average properties of EV subtypes, making it difficult to precisely characterize individual particles. Therefore, it is imperative to assess the behaviors of individual EV particles. In this review, we present a comprehensive overview of recent imaging studies on individual EV particles, including single-fluorescent-particle tracking, single-metal-nanoparticle tracking, single-non-label-particle tracking, super-resolution microscopy, and atomic force microscopy (AFM).

## 2. Single-Fluorescent-Particle Tracking

**Figure 1:**
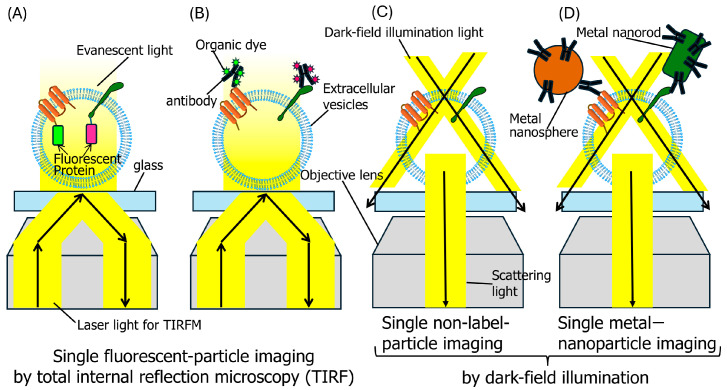
The imaging of individual EVs. (**A**) The single-fluorescent-particle imaging of EVs. Molecules are labeled with fluorescent proteins or organic dyes via tags. The dye-to-protein ratio is 1. (**B**) The single-fluorescent-particle imaging of EVs. Molecules are labeled with organic dye-conjugated antibodies. The number of dye molecules conjugated to antibodies follows a Poisson distribution. (**C**) The single-non-label-particle imaging of EVs via dark-field illumination. (**D**) The single-metal-nanoparticle tracking of EVs via dark-field illumination.

To identify EV subtypes and determine the specific components contained within each subtype, single-fluorescent-particle tracking (SPT) has been employed using total internal reflection fluorescence microscopy (TIRFM) (Figure 1) [28,29,30,31,32,33]. Initially, EVs have to be labeled with fluorophores. While single EV particles have been visualized by incorporating lipid-like probes into EVs, this approach does not enable the observation of individual EV subtypes. Additionally, because lipid-like probes can integrate into molecular assemblies lacking a lipid bilayer structure [34], such as exomeres, it is essential that EVs are thoroughly purified before fluorescent labeling. Numerous EV marker proteins have been identified, with the most established examples being the tetraspanins CD63, CD81, and CD9. These proteins form complexes with integrins and growth factors, modulating their functions. One method for fluorescent labeling of EV marker proteins is to use fluorescently labeled antibodies after EV purification (Figure 1B).

Han et al. developed a method to immobilize EVs using biotinylated antibodies against tetraspanins bound to a DDS–tween 20-coated cover glass via avidin and biotinylated bovine serum albumin, allowing for the observation of individual EV particles after labeling with fluorescently labeled secondary antibodies [31]. They performed dual-color imaging by employing two different antibodies against tetraspanins and two distinct fluorescently labeled secondary antibodies. This system was highly effective at detecting EV subtypes, as the non-specific binding of EVs to glass surfaces was significantly reduced by the DDS and tween 20 coatings. Their findings revealed that EVs were derived from HEK293.

MCF-7 and B16BL6 cells all exhibit subtypes containing all three tetraspanins at fractions of 11%, 10%, and 1%, respectively. Moreover, over 50% of EVs contained only CD63, CD81, or CD9. However, in this system, quantifying the exact number of tetraspanins was not feasible, as the dye-to-protein ratio of the fluorescent secondary antibodies used ranged from 2 to 8, and dyes were conjugated to antibodies according to a Poisson distribution. It would be intriguing to observe EVs using TIRFM with single-molecule sensitivity in the future to quantify the number of proteins per EV. To achieve this objective, proteins should be fluorescently labeled with fluorophores via tags at a 1:1 ratio (Figure 1D, Table 1). Meanwhile, Mizenko et al. reported that detection sensitivity varies significantly between antibodies, complicating the expression level comparison (Table 1) [35]. They also noted that the binding of antibodies to tetraspanins can be suppressed by steric hindrance and/or a limited number of binding sites being available. Consequently, these findings suggest that experiments using antibodies to determine tetraspanin colocalization on EVs should be performed with great caution.

On the other hand, two types of fluorescently tagged tetraspanins can be co-expressed in cells to perform two-color observation [30]. While this method allows for quantitative analysis, caution must be exercised when interpreting the results. The overexpression of fluorescently labeled marker proteins may lead to their localization in sEVs where they are not normally present. Additionally, to accurately represent the relationship between marker molecules, it is essential to use stable co-expression systems where the expression levels of tagged proteins are within an appropriate range relative to endogenous molecules. Moreover, it is important to incorporate fluorescently labeled marker molecules into sEVs, which reflect those of endogenous proteins. For example, a recent study on the contents of various marker proteins fused with GFP in sEVs revealed that GFP-fused ALIX and flotillin-2 are rarely present in EVs, whereas tetraspanins, such as GFP-fused CD63, CD9, and CD81, are efficiently incorporated into EVs (Figure 1, Table 1) [30].

**Figure 2:**
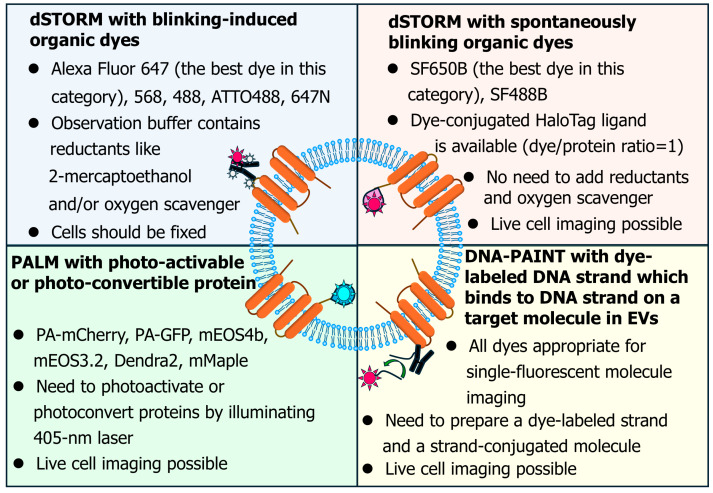
The application of super-resolution single-molecule localization microscopy (SMLM) to investigate the characteristics of EVs such as the size and number of molecules.

Nucleic acids encapsulated within EVs have also been observed at the single-molecule level [55]. Using fluorescently labeled nucleic acid probes to complement specific sequences, single-molecule fluorescence in situ hybridization (smFISH) enables RNA visualization and quantification at the single-molecule level [56]. The binding of multiple probes generates a detectable fluorescent signal at a diffraction-limited spot, captured using conventional fluorescence microscopy. Furthermore, recent EV studies have employed the single-particle interferometric reflectance imaging sensor (SR-IRIS), a technique that combines interferometric imaging with fluorescence microscopy, enabling the simultaneous profiling of the size, concentration, and protein biomarker analysis of individual biological particles within a bulk population [57,58]. Troyer et al. developed a SPIRFISH method, integrating smFISH with SR-IRIS to allow fluorescence-based HIV-1 genomic RNA detection within individual virions [59]. They successfully distinguished infectious HIV virions from host-derived EVs by co-detecting HIV Env g120 and HIV genomic RNA in single particles with a characteristic diameter.

## 3. Single-Metal-Nanoparticle Tracking

Besides fluorescent dyes, metal nanoparticles can also be used for labeling EVs, since metal nanoparticles can be observed via light scattering in a dark field (Figure 1, Table 1). Dark-field microscopy employs a dark-field condenser lens to direct a cone of light away from the objective lens (Figure 1C,D). Only the light scattered by the samples enters the objective lens, enabling the observation of samples with high contrast and minimal background illumination. Metal nanoparticles are effective tools for visualizing various biomolecules and biological structures, including proteins, lipids, and cellular organelles, via optical microscopy, especially dark-field microscopy [60]. Therefore, individual EV particles labeled with metal nanoparticles can be continuously monitored without photobleaching. The metal nanoparticle’s position is determined by locating the center of a bright spot in the optical image, with the accuracy inversely proportional to the photon count’s square root [61]. In other words, a high photon count is essential for precise measurements. Given their strong light-scattering properties that result from plasmon resonance, gold nanoparticles enable sub-millisecond temporal resolution and nanometer-scale positional accuracy [60]. Liang et al. performed a nanoplasmon-enhanced scattering (nPES) assay using antibody-conjugated gold nanospheres and nanorods to target EVs captured by EV-specific antibodies on a sensor chip, generating a local plasmon effect that enhanced sensitivity and specificity when detecting tumor-derived EVs. The gold nanospheres were 50 nm in diameter, while the gold nanorods had dimensions of 25 × 60 nm [36]. Gold nanoparticles scatter light at specific wavelengths determined by their size and shape. In [36], the gold nanospheres and nanorods scattered green and red light, respectively. When the distance between the nanospheres and nanorods was less than 200 nm, their scattering coupled, creating a plasmon that shifted the scattering spectrum to yellow and significantly increased scattering intensity. Using this method, the authors identified ephrin type-A receptor 2 (EphA2) as a biomarker for pancreatic cancer EVs and demonstrated that an nPES assay for EphA2-EVs could distinguish pancreatic cancer patients from those with pancreatitis and healthy individuals. In addition, Amrheim et al. developed a simple method to detect surface proteins on individual gold nanoparticles that employed a dual imaging approach [37]. Using this method, EVs were labeled with cholesterol-PEG-Cy5 and captured on a multi-well gold chamber slide via CD81 antibodies. Specific surface proteins on EVs were then labeled with antibody-conjugated 60 nm gold nanoparticles and detected through dark-field imaging. The imaging of both EVs and their surface proteins was achieved using a dual fluorescence and dark-field imaging system at the single-EV and single-particle levels [37,38]. Furthermore, Amrollahi et al. developed a far-field nanoplasmon-enhanced scattering (FF-nPES) assay for the isolation-free characterization of EVs in small serum volumes (<5 μL) [39]. EVs were captured using a cancer-selective antibody, hybridized with gold nanorods conjugated to an antibody targeting the EV surface protein CD9, and quantified based on their light scattering properties when analyzed using a fully automated dark-field microscopy system. Their results indicated that FF-nPES’s performance was comparable to that of EV ELIZA when analyzing the surface expression of epithelial cell adhesion molecules on EVs. Observing EVs and their constituent proteins tagged with nanospheres and nanorods using dark-field imaging is a powerful technique for evaluating the molecular composition of EVs. However, we must remember that metal nanoparticles are larger than fluorescent molecules, and the use of antibodies against EV proteins for nanoparticle labeling may introduce steric hindrance, as mentioned in the previous section.

## 4. Single-Non-Label EV Particle Tracking

Fluorescent labeling can be troublesome and may negatively affect molecules’ incorporation into EVs, as mentioned earlier. Additionally, the photobleaching of fluorescent dyes must be considered during analysis. To address these challenges, label-free optical imaging techniques have been employed. Nanoparticle tracking analysis (NTA) using dark-filed imaging has frequently been used to perform quantitative assessments of size and refractive indexes, and numerous studies have utilized NTA to measure EV sizes (Figure 1, Table 1) [40,41]. However, since NTA does not distinguish between EV subtypes, it only provides the average size of the total EV population. Furthermore, because EVs are extremely small, label-free NTA tends to be less sensitive. To overcome this limitation, recent studies developed improved dark-field imaging techniques. Chen et al. introduced a reflective surface, enhancing dark-field scattering microscopy (REDFSM) sensitivity, allowing for the deep tracking of single EVs in free solution, as well as the clear observation and identification of organelles in single cells, with minimal exogenous interference [42]. In REDFSM, incorporating a reflective surface beneath the particles enables peripheral incident light to illuminate the particle after reflection, thereby enhancing the scattering intensity. In addition, Wang et al. observed single EV particles on a chip using light-sheet illumination, effectively reducing background noise [43]. Single EV particles were tracked using a deep-learning algorithm. In brief, a simulated image, closely matching the experimental results, was employed as a training input. Small EVs (sEVs) from normal and cancerous liver cell lines, as well as those from sorafenib-treated cancerous cells, were analyzed. The authors found that three types of sEVs could be well differentiated based on their particle size distribution. More recently, a label-free nanoparticle diffusion analysis method was developed, utilizing light scattering imaging and dark-field technology to accurately measure EVs’ size and diffusion coefficients [44]. By applying sliding window analysis and principal component analysis [62], the trajectories of Brownian diffusion and non-Brownian diffusion were discriminated, and EV sizes were estimated from the diffusion coefficients of Brownian diffusion only. The EVs from cancerous plasma, isolated from mice after injection with liver cancer cells (hepatoma cells), were significantly larger than those obtained from normal plasma.

## 5. Super-Resolution Microscopy

Single-molecule localization microscopy (SMLM) allows for the determination of structural contours at a spatial resolution of 10–20 nm. Techniques such as direct stochastic optical reconstruction microscopy (dSTORM) and photoactivated localization microscopy (PALM) have been used to measure EV diameters and detect small domains within EVs (Figure 2, Table 1). For dSTORM and PALM imaging, molecules within EVs must be labeled with blinking dyes at an appropriate density, as overlapping fluorescent spots hinder tracking, while insufficient spot localization reduces spatial resolution. When employing blinking-induced organic dyes such as Alexa647 and ATTO647N, reductants like 2-mercaptoethanol and oxygen scavenger should be included in the observation buffer to induce dye blinking, and cells should be fixed before observation. In contrast, if spontaneously blinking dyes such as SF650B [63] are used for dSTORM imaging [64], adding reductants and oxygen scavengers is unnecessary, allowing for live cell imaging. For PALM imaging, photoactivable or photoconvertible proteins are utilized, activated by 405 nm laser illumination, enabling live cell imaging. Point accumulation for imaging in nanoscale topography (PAINT) is also advantageous for super-resolution microscopy in the characterization of EVs [65]. To perform DNA-PAINT, one of the most frequently used PAINT techniques, an organic dye-conjugated DNA strand must be prepared, and the target molecule must be tagged with a complementary DNA strand (Figure 2). Interaction between these DNA strands should be transient, allowing the fluorescent spot on the target molecule to appear briefly. McNamara et al. performed three-dimensional dSTORM and PALM observations of EVs containing CD81-mCherry, CellMask Red (CM Red), and CD9 stained with Alexa488 anti-CD9 antibody [45]. The average diameter of isolated EVs was 126 nm, and membrane microdomains were present within EV membranes. This research was a cutting-edge imaging study that provided insights beyond EV sizes, although the further optimization of fluorophores and observation buffers may be required for enhanced super-resolution microscopy. Lennon et al. performed quantitative single-molecule localization microscopy (qSMLM) on EVs derived from PANC-1 cells (pancreatic cancer cells) affinity-isolated using the clinical antibody cetuximab against EGFR [28]. They also used cetuximab to immobilize EVs on a glass surface and labeled them with Alexa647-cetuximab. Using qSMLM, they determined the EV size to be 51 nm and quantified the number of EGF receptor (EGFR) molecules per EV to be 28. Additionally, they found that EVs from pancreatic ductal adenocarcinoma (PDAC) patients exhibited multiple populations, with the PDAC-associated population containing larger EVs but fewer EGFR and CA19-9 molecules. Therefore, the qSMLM of EVs shows potential as a sensitive PDAC detection method. In a related study, Jiang et al. (the same group as Lennon et al. [28]) reported that HER2-enriched EVs from plasma from breast cancer patients had distinct characteristics, including a typically higher number of tetraspanin molecules and a larger size [46].

Another notable super-resolution microscopy technique, stimulation emission depletion (STED) microscopy, enables the observation of cellular structures at spatial resolutions as small as 30 nm. Dechantsreiter et al. observed CD81 stained with Alexa647-conjugated anti-CD81 antibodies via confocal microscopy and actin stained with Alexa488-phalloidin via STED microscopy in fixed macrophages [66]. They found that EVs containing CD81 were segregated from the actin cytoskeleton in IgG-activated M0-, M1-, and M2-like macrophages. Forero et al. recently performed STED microscopy to investigate the uptake of EVs by neurons after fixation with 4% paraformaldehyde, revealing that the EV cellular localization in recipient cells was distinctly cell-type-specific [67]. Additionally, in another study, STED microscopy visualized the pH indicator acridine orange [68]. Thus, both SMLM and STED microscopy have been increasingly applied for individual EV particle and cellular structure observation after chemical fixation.

## 6. Atomic Force Microscopy

Atomic force microscopy (AFM) is a powerful tool for evaluating the surface properties of biological samples with spatial resolutions of less than 2 nm, and it has been used to characterize EVs (Table 1) [47,69]. Before AFM analysis, EVs must be immobilized on a surface (Table 1). Muscovite mica is often the preferred choice for immobilization because it can be easily cleaved, leaving a negatively charged, atomically flat surface, and it is commonly used as a substrate for the single-molecule imaging of DNA and proteins [47]. AFM measures not only the size distribution but also the mechanical properties of EVs [48,49]. These quantitative measurements are crucial, as the mechanical properties of EVs significantly influence their biological function. Ridolfi et al. performed the high-throughput nanomechanical screening of single EVs using AFM [50]. They captured a nanomechanical snapshot of an EV sample, allowing them to discriminate between subpopulations of vesicular and non-vesicular objects in the same sample, as well as between populations of vesicles with similar sizes but different mechanical properties. Using this technique, Ridolfi et al. successfully determined the relative abundance of EVs and lipoproteins in mixed samples [51]. Sajidah et al. employed high-speed AFM to evaluate nanotopological changes in EVs under different physicochemical stresses, including thermal, pH, and osmotic stresses [70]. They observed irreversible structural changes in EVs during pH or osmolarity transition. Furthermore, AFM has been employed for the functional analysis of protein quantities in EVs using antibody-coated tips [52]. Gazze et al. employed PeakForce-Quantitative Nano Mechanics (PF-QNM) to obtain structural insights into EVs following their purification and downstream processing [54]. PF-QNM is non-destructive to both the tip and the sample, directly controlling the peak normal force and minimizing lateral forces on the probe. This technique enables the mapping and differentiation of nanomechanical properties with atomic resolution via topographical imaging. Using PF-QNM, the authors demonstrated that EV ultracentrifugation slightly increased their physical dimensions and reduced EV adhesion, accompanied with a decrease in CD63 content. Sonicated EVs exhibited significantly lower levels of CD81, a reduced size, increased Young’s modulus, and diminished adhesive force. These biomechanical and biochemical alterations underscore EV sample preparation techniques’ impacts on the critical properties associated with EV biological functions. Recently, combining AFM with other imaging techniques has enabled the discovery of new EV profiles. For example, using AFM IR spectroscopy (AFM-IR) [71], Kim et al. studied the structural compositions of single EVs, revealing heterogeneity across individual EVs [53]. The AFM-IR technique employs photothermal-induced resonance (PTIR). When the sample is irradiated with infrared laser light, molecular vibrations generate localized heating, leading to the significant thermal expansion of the sample. This rapid expansion, induced by the adsorption of nanosecond pulses of infrared light, is detected as a ringdown signal by the highly sensitive AFM cantilever. As the ringdown amplitude is directly proportional to the infrared irradiation dose, the AFM-IR spectrum is obtained by plotting the Fourier-transformed amplitude values against the irradiated infrared laser light’s wavelength.

## 7. Discussion and Future Directions

Various imaging techniques have been developed to evaluate individual EV particles. Recent advances in these methods have enabled the high-throughput analysis of the membrane composition, size, and physical properties of individual EVs. However, as mentioned, individual EV particle and cellular structure imaging is often performed with immunostaining in cells after chemical fixation. We must note that immunostaining after chemical fixation can alter cellular structures, particularly those composed of lipid-anchored receptors and signaling molecules [72]. After fixation with 4% paraformaldehyde for 15–30 min, many lipids and lipid-anchored proteins remain mobile, and antibodies used for staining can crosslink these mobile molecules in membranes. EV membranes are abundant in a liquid-ordered (Lo)-like phase [73], as well as enriched with raftophilic molecules, including glycosphingolipids [74] and glycosylphosphatidylinositol (GPI)-anchored proteins [75,76]. Consequently, live-cell imaging would be preferable for visualizing individual EV particles and cell structures in their native states.

**Figure 3:**
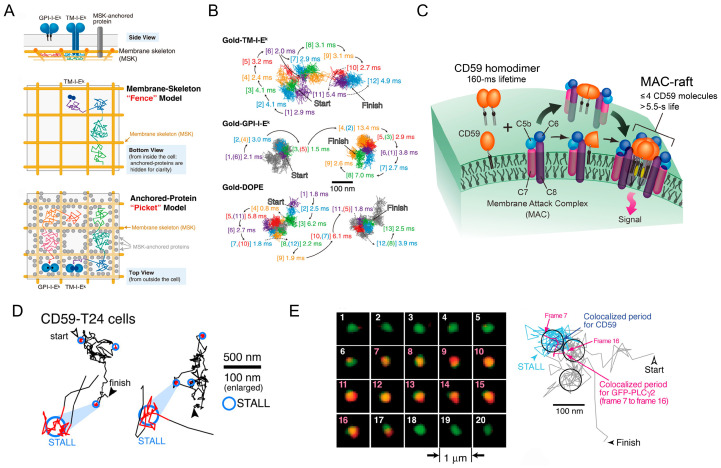
Single-molecule imaging is a powerful tool for unraveling mechanisms of stochastic and/or rare events. (**A**) A schematic diagram of the fence model and the picket model. Transmembrane proteins (TM-I-E^k^) undergo hop diffusion due to steric hindrance by membrane cytoskeletal proteins in the cell plasma membrane (fence model; middle), while GPI-anchored proteins undergo hop diffusion due to transmembrane proteins (pickets) anchored to membrane cytoskeletal proteins (picket model; bottom). (**B**) The representative trajectories of a transmembrane protein (TM-I-E^k^), a GPI-anchored protein (GPI-I-E^k^), and a control phospholipid (DOPE) tagged with 40 nm colloidal gold particles, observed at a 25 μs/frame. (**C**) A schematic diagram of the organization of the GPI-anchored protein CD59. CD59 formed transient homodimers in the steady state, but formed stable homo-oligomers upon stimulation with the ligand (membrane attack complex or MAC). (**D**) Representative trajectories of CD59 upon stimulation. CD59 exhibited the stimulation-induced temporal arrest of lateral diffusion (STALL), as shown in the blue circle. (**E**) Left: a representative image sequence of a CD59 oligomer (green) and a single molecule of GFP-PLCγ2 (red), observed at a video rate. The numbers 1–20 mean frame numbers. Right: trajectories of a CD59 oligomer and a single molecule of GFP-PLCγ2. A single molecule of PLCγ2 was recruited to a CD59 oligomer during the STALL (frame 7 to 16). Adapted from Figure 1 and Figure 6a in [77], Figure 7d in [80], Figure 4, and Figure 5a,b in [85] with permission.

Live-cell imaging is essential for several additional reasons. Molecules within cells undergo thermal diffusion and do not collide synchronously, making molecular interactions inherently stochastic. In other words, cellular molecules function in a fundamentally heterogeneous manner. Furthermore, typically, only small fractions of cellular molecules are involved in interactions with other molecules. For example, GPI-anchored receptors, representative of raft molecules and enriched in EV membranes [75,76], exhibit confined diffusion in small PM domains for short periods before suddenly hopping across compartment boundaries, where they are once again confined in small domains (Figure 3A,B). These hopping events occur asynchronously, driven by the membrane skeletal fence and anchored protein picket structures [77]. GPI-anchored receptors form small clusters induced by transbilayer lipid coupling in the PM [78,79]. GPI-anchored proteins usually form transient homodimers (~150 ms) (Figure 3C), but only a fraction of these receptors form homodimers, and the timing of these events is random [80]. These homodimers of GPI-anchored receptors recruit other raftophilic lipids, such as gangliosides [81] and sphingomyelin [82], thereby inducing small Lo-like domain formation [83,84]. Upon ligand activation, GPI-anchored receptors form stable oligomers (Figure 3C), inducing temporal confinement in small membrane domains, known as the stimulation-induced temporary arrest of lateral diffusion (STALL) (Figure 3D), ultimately leading to the recruitment of intracellular signaling molecules such as PLCγ2 (Figure 3E) [85]. These stochastic and/or rare events occur in various receptors, including receptor tyrosine kinases such as EGF receptors [86,87,88], G protein-coupled receptors [89,90,91], neurotransmitter receptors such as AMPA receptors [92,93,94], cytokine receptors [95,96,97], immunoreceptors such as T-cell receptors [98,99,100,101,102,103,104], and adhesion molecules such as integrin [105,106,107] in the PM, as well as STING [64] in Golgi membranes and VAPB in ER membranes [108]. These stochastic and/or rare events’ dynamics can only be visualized by single-fluorescent-molecule imaging (SFMI). Therefore, SFMI via TIRFM is a powerful tool for detecting such events in living cells, providing critical insights into underlying mechanisms, including individual EVs’ behaviors.

**Figure 4:**
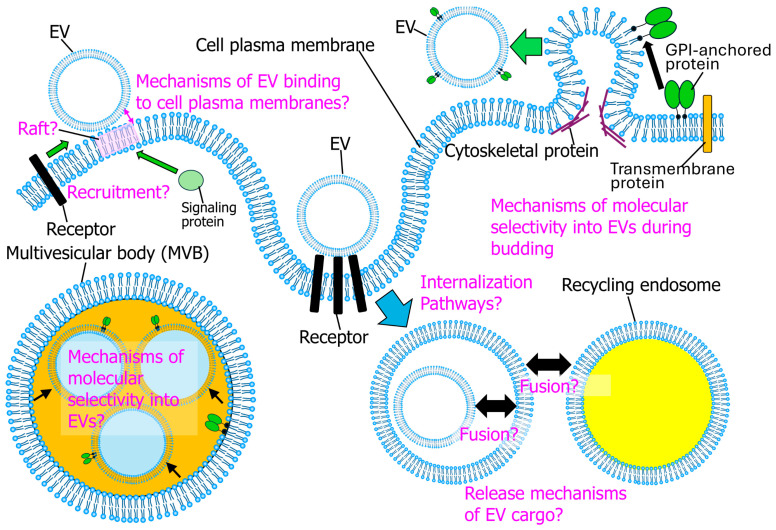
The remaining issues concerning EV interactions with cellular structures that must be resolved via single-molecule imaging and super-resolution microscopy.

The simultaneous observation of single EV particles and super-resolution imaging of cellular structures in fixed cells has only recently begun [66]. However, to the best of our knowledge, the SPT of EVs and SFMI of cellular molecules has never been simultaneously performed in living cells. As noted, cellular molecules behave in an inherently heterogeneous manner, with only a small fraction of molecules participating in molecular interactions upon stimulation. Many unresolved issues remain concerning EVs’ interactions with donor and recipient cells (Figure 4). For example, the mechanisms via which molecules are selectively partitioned into EVs in the multivesicular bodies (MVBs) of donor cells and molecules are partitioned into EVs budding from the plasma membrane are not yet fully elucidated. Three-dimensional single-molecule imaging is a valuable tool for investigating membrane protein localization during EV budding. During the release of EVs from the tip of the PM protrusion, the simultaneous observation of membrane and cytoskeletal proteins can elucidate why GPI-anchored proteins are readily incorporated into EVs, whereas only specific transmembrane proteins are incorporated (Figure 4). The membrane skeletal fence and anchored protein picket structures, as shown in Figure 3A,B, may be crucial for this process. In addition, single-molecule imaging enables the exploration of the relationship between the degree of oligomerization of GPI-anchored proteins and their incorporation into EVs, as shown in experiments analogous to Figure 3C. Although it has been suggested that EV binding to the plasma membranes of recipient cells is facilitated by interactions between integrin subunits in EVs and extracellular matrix (ECM) components such as fibronectin and laminin [25], this binding has not been directly investigated. Single-particle observations of sEVs and super-resolution microscopy of the ECM may resolve this issue. Furthermore, single-molecule imaging of interactions between EVs and membrane molecules or cytosolic signaling proteins in the PMs of recipient cells (as shown in Figure 3D,E) could elucidate whether the membrane domains beneath sEVs are lipid rafts and uncover the mechanisms through which EVs are transported into the PM’s uptake machinery. Moreover, the EV internalization pathways used by the recipient cells have not been thoroughly examined. The simultaneous observation of individual EV particles and specific uptake machineries like caveolae and clathrin-coated pits may resolve this issue. Additionally, the process through which EV cargos such as miRNA are released into recipient cell cytosol remains elusive. The efficiency of fusion between EVs and endocytic vesicles, as well as between endocytic vesicles and recycling endosomes, is not precisely known yet. Single-molecule imaging and super-resolution microscopy may resolve this issue. Thus, studies using single-molecule and super-resolution imaging techniques in living cells to explore EV interactions with cellular structures are in the early stage, and high-resolution imaging presents a promising avenue for future research.

## Figures and Tables

**Table 1:** Imaging techniques of individual EV particles.

Methods	Illumination	Label	Advantage	Disadvantage	Refs.
Single-fluorescent-particle tracking	TIRF or oblique angle illumination	Fluorescent proteins or organic dyes	Dye/protein = 1 EV subtypes are distinguishable	Photobleaching, difficult for some proteins to partition into EVs	[30]
		Dye-conjugated antibody	Easy to perform; EV subtypes are distinguishable	Various dyes/proteins, steric hindrance, photobleaching	[28,31,32,33,35]
Single-metal-nanoparticle tracking	Dark-field illumination	Metal particles or nanorods	No photobleaching	Steric hindrance	[36,37,38,39]
Single-non-label-particle tracking	Dark-field illumination	Non-label	No photobleaching	EV subtypes are indistinguishable	[40,41,42,43,44]
Single-molecule localization microscopy (also see Figure 2)	TIRF or oblique angle illumination	Photoactivable fluorescent proteins, blinking organic dyes	Possible to determine the EV size and the number of molecules per EV	It takes a long time to acquire one image	[28,45,46]
Atomic force microscopy	Use tip and cantilever	Non-label	High spatial resolution (better than 2 nm)	Need to be immobilized on a substrate	[47,48,49,50,51,52,53,54]

## Data Availability

No new data were created or analyzed in this study. Data sharing is not applicable to this article.
